# Overexpression of the Heterochromatinization Factor BAHD1 in HEK293 Cells Differentially Reshapes the DNA Methylome on Autosomes and X Chromosome

**DOI:** 10.3389/fgene.2015.00339

**Published:** 2015-12-01

**Authors:** Emanuele Libertini, Alice Lebreton, Goran Lakisic, Marie-Agnès Dillies, Stephan Beck, Jean-Yves Coppée, Pascale Cossart, Hélène Bierne

**Affiliations:** ^1^Plate-forme Transcriptome et Epigénome, Département Génomes et Génétique, Institut PasteurParis, France; ^2^Medical Genomics Group, UCL Cancer Institute, University College LondonLondon, UK; ^3^Unité des Interactions Bactéries-Cellules, Institut PasteurParis, France; ^4^Institut National de la Santé et de la Recherche Médicale U604Paris, France; ^5^Institut National de la Recherche Agronomique USC2020Paris, France; ^6^Institut National de la Recherche Agronomique, UMR1319 MICALISJouy-en-Josas, France; ^7^AgroParistech, UMR MICALISJouy-en-Josas, France

**Keywords:** cytosine methylation, DNA methylation, whole genome bisulfite sequencing, heterochromatin, epigenetics, LAD, Xi

## Abstract

BAH domain-containing protein 1 (BAHD1) is involved in heterochromatin formation and gene repression in human cells. BAHD1 also localizes to the inactive X chromosome (Xi), but the functional significance of this targeting is unknown. So far, research on this protein has been hampered by its low endogenous abundance and its role in epigenetic regulation remains poorly explored. In this work, we used whole-genome bisulfite sequencing (BS-seq) to compare the DNA methylation profile of HEK293 cells expressing low levels of *BAHD1* (HEK-CT) to that of isogenic cells stably overexpressing *BAHD1* (HEK-BAHD1). We show that increasing BAHD1 levels induces *de novo* DNA methylation on autosomes and a marked hypomethylation on the X chromosome (chrX). We identified 91,358 regions that have different methylation patterns in HEK-BAHD1 compared to HEK-CT cells (termed “BAHD1-DMRs”), of which 83,850 mapped on autosomes and 7508 on the X chromosome (chrX). Autosomal BAHD1-DMRs were predominantly hypermethylated and located to satellites, interspersed repeats, and intergenic regions. In contrast, BAHD1-DMRs on chrX were mainly hypomethylated and located to gene bodies and enhancers. We further found that BAHD1-DMRs display a higher-order organization by being clustered within large chromosomal domains. Half of these “BAHD1-Associated differentially methylated Domains” (BADs) overlapped with lamina-associated domains (LADs). Based on these results, we propose that BAHD1-mediated heterochromatin formation is linked to DNA methylation and may play a role in the spatial architecture of the genome.

## Introduction

DNA methylation is a heritable epigenetic mark that has critical roles in the regulation of genome structure and transcription in eukaryotes (for reviews see Suzuki and Bird, 2008; Laird, 2010). This modification, catalyzed by DNA methyltransferases (DNMTs), is essential for embryonic development and a number of key processes, such as X chromosome inactivation, genomic imprinting, chromosome stability, and silencing of repetitive elements (Li et al., 1993; Jones and Laird, 1999; Baylin et al., 2001; Geiman and Muegge, 2010). Consistent with these important roles, changes in DNA methylation are associated with several human diseases (Robertson, 2005).

In somatic mammalian cells, DNA methylation mainly occurs at CpG dinucleotides, with ~70% of CpG being methylated (mCpG; Lister et al., 2009). This modification is dynamically regulated in a developmental context across multiple cell types (Ziller et al., 2013) and is commonly associated with gene silencing, especially within CpG islands (CGIs). CGIs in promoters are predominantly unmethylated and can be methylated in a tissue-specific manner (Straussman et al., 2009; Deaton and Bird, 2011). CGI methylation contributes for instance to the mechanism of X chromosome inactivation (Sharp et al., 2011), which results in one inactive X chromosome (Xi) and one active X chromosome (Xa) in mammalian female cells (Nora and Heard, 2010; Pollex and Heard, 2012). Methylation of CGI promoters on Xi, in contrast to Xa, correlates with silencing of ~85% of all Xi genes (Carrel and Willard, 2005; Zhang et al., 2013). However, several studies indicate that DNA methylation is not only functionally linked to gene repression (Suzuki and Bird, 2008; Ndlovu et al., 2011). For instance, a positive correlation between active transcription and gene body methylation has been detected on the Xa and evidence suggest that X-linked gene bodies are less methylated on Xi than on Xa (Viegas-Pequignot et al., 1988; Prantera and Ferraro, 1990; Weber et al., 2005; Hellman and Chess, 2007). A link between active transcription and gene body methylation has also been detected at highly expressed genes on autosomes (Aran et al., 2011). Conversely, in cancer cells, formation of repressive domains coincides with global hypomethylation (Hon et al., 2012).

Mechanisms controlling which DNA sequences become methylated involve recruitment of DNMTs at specific loci, though the driving mechanism is not well understood. There is a complex interplay between DNA methylation and histone modifications. This relationship can be partially mediated by interactions of DNMTs with histone lysine methyltransferases (KMT, such as SETDB1, SUV39-H1, and G9a) and by action of mCpG-binding proteins, such as MECP2 and MBDs, that are capable of recruiting histone deacetylases (HDACs) to the methylated region (for reviews see Bernstein et al., 2007; Cedar and Bergman, 2009; Buck-Koehntop and Defossez, 2013). However, how this scenario unfolds at specific genome loci according to time, cell type and stimuli, is not well characterized. Hence, it is crucial to identify components of chromatin-remodeling complexes that bridge DNA methylation, histone modifications and transcription factors.

One such component could be BAHD1, a vertebrate-specific protein that we previously identified as a repressor involved in chromatin compaction (Bierne et al., 2009). Overexpression of BAHD1 in human cells is sufficient to stimulate *de novo* formation of heterochromatic foci that lack acetylated histone H4. Also, exogenous BAHD1 localizes to the heterochromatic Xi in female cell lines. This heterochromatin factor co-purifies with HDAC1/2, HP1 (α, β, γ), and KAP1/TRIM28 (Lebreton et al., 2011) and interacts with the methyl-CpG-binding protein MBD1 and the KMTs SETDB1 (Bierne et al., 2009) and SUV39-H1 (Weimann et al., 2013) suggesting that it belongs to a multiprotein chromatin-repressive complex (“the BAHD1 complex”; Bierne et al., 2012). Several BAHD1-associated partners are known to interact with DNMT3A (Fuks et al., 2001, 2003; Lehnertz et al., 2003; Li et al., 2006) and KAP1 plays a role in DNA methylation (Quenneville et al., 2011). Collectively, these findings suggest an attractive hypothesis wherein BAHD1 plays a role in the relationship between DNA methylation and chromatin compaction in higher eukaryotes. However, research on this protein has been hampered by its absence or low abundance in mammalian tissues, making it difficult to study its effect on the epigenome.

In this study, we searched for a connection between BAHD1 and DNA methylation by using, as an experimental model, Human Embryonic Kidney 293 (HEK293) cells stably expressing BAHD1. In the widely used HEK293 line, our previous studies showed that BAHD1-associated heterochromatin and multiprotein complexes are formed when *BAHD1* expression increases (Bierne et al., 2009; Lebreton et al., 2011). Using whole genome bisulfite sequencing (BS-seq; Lister et al., 2009), we generated methylomes at single-nucleotide resolution of HEK293 cells with endogenous or stable overexpression of *BAHD1*. Comparison of the methylation profiles of these two lines suggests that BAHD1 contributes to the differential patterning of DNA methylation, both at local (kb) and large (Mb) genome scales, with distinct effects on autosomes and on the X chromosome (chrX). In addition, gene expression data suggest that BAHD1-associated differential methylation could be associated with transcriptional repression.

## Materials and methods

### Cell lines, plasmids, antibodies, and immunostaining

The stable cell line with constitutive *BAHD1* expression (HEK-BAHD1) and its isogenic control (HEK-CT) are described in the Supplementary Material. The HPT-BAHD1 inducible cell line and isogenic HPT-HEK293 control are described in Lebreton et al. (2011). Cells were grown at 37°C in a humidified 10% CO_2_ incubator, in Dulbecco's modified Eagle's medium with GlutaMAX TM (Gibco) supplemented with 10% FBS (Gibco) and Hygromycin (HygroGold, Invivogen 200 μg/ml). To induce *BAHD1* expression in HPT-BAHD1 cells, tetracycline was added to cell culture media (11 μg/ml) 30 h before cell recovery. Plasmid pYFP-BAHD1, which was used for transient overexpression of BAHD1-YFP, is described in Bierne et al. (2009). Antibodies used in the study were raised against BAHD1 (Abcam, 46573) and HDAC1 (Abcam, ab7028). Preparation of chromatin extracts is described in Lebreton et al. (2011). Immunofluorescence and XIST FISH assays were carried out as described in Bierne et al. (2009).

### BS-seq procedures

Isolated DNA was truncated into 100–300 bp fragments by sonication followed by DNA-end repair, 3′-dA overhang addition and ligation of methylated sequencing adapters. Samples underwent bisulfite treatment with the ZYMO EZ DNA Methylation-Gold kit. Desalted, size-selected, PCR amplified fragments were size-selected again. Quality controlled libraries were selected for Illumina sequencing by the Beijing Genomics Institute (BGI). Data filtering included removing adaptor sequences, contamination and low-quality reads from raw reads. These reads were filtered by custom BGI scripts, as described in Li et al. (2010). There were two types of low-quality reads removed from the data: (1) when the ratio of N in whole read was over 10%; (2) when the ratio of base whose quality was < 20 was over 10%. Observed cytosines on the forward read of each read pair were *in silico* replaced by thymines, and observed guanines on the reverse read of each read pair were *in silico* replaced by adenines (Xiang et al., 2010). The “alignment form” reads were then mapped to the “alignment form” reference genome by *SOAP aligner*. Every hit with a single placement with minimum numbers of mismatches and a clear strand assignment was defined as unambiguous alignment (uniquely mapped reads) and was used for methyl-cytosine determination. Only the uniquely mapped reads were used to estimate the copy numbers of the local region (Xiang et al., 2010). Genomic bases with a copy number larger than 1.5 were not used to call methylcytosines and not used in any subsequent analysis to avoid errors caused by misalignment following the protocol described in Li et al. (2010). For each of the replicates, 1.03 billion (HEK-CT) and 1.05 billion (HEK-BAHD1) paired-end reads of 90 bp length were generated and aligned to the female human reference sequence (NCBI build 37/hg19), yielding 93.0 and 94.3 gigabases (Gb), with an average of 23.4 and 26.1-fold genome coverage. In total, ~38 and ~37 million methylcytosines were detected in HEK-CT and HEK-BAHD1, respectively, where >95% of the queried CpGs had at least one read, and ~85% had more than four reads. Methylcytosines amounted for 3.35 and 3.45% of the cytosines with sequence coverage and ~97% of them were in the CpG context. Rare non-CpG methylation (CHH and CHG) was present throughout all chromosomes (Supplementary Figure S1). Whole-genome BS-seq reads were subjected to data import, smoothing, and DMR analysis with the Bioconductor package *bsseq* (Hansen et al., 2012). Replicates were tiled over 300 bp windows for statistical analysis where Fisher's exact test was applied for inference with multiple-testing adjusted values (Benjamini-Hochberg procedure, *p* < 0.1). DMRs were defined by subsetting the results based on a regional methylation difference of at least 25% across the 300 bp windows. To investigate a higher order organization, BAHD1-specific DMRs were first counted and grouped over windows of 0.5 Mb, defining “BAHD1-DMR clusters.” Then, the mapping of DMR-rich regions was refined by defining contiguous domains of DMR clusters with more than the 75% quantile of DMR counts (i.e., representing the top 25% quantile). These domains were referred to as “BAHD1-Associated differentially methylated Domains” (BADs). The overlap between BADs and LADs was calculated in the R environment using the data from Guelen et al. (2008) accessible via the UCSC genome browser. Full datasets have been deposited in Gene Expression Omnibus (GEO, http://www.ncbi.nlm.nih.gov/geo) and are accessible through GEO series accession number GSE51867.

### Native ChIP-seq procedures

The immunoprecipitation of the HPC-BAHD1 protein in HPT-BAHD1 cells with anti-Protein C antibodies is described in Lebreton et al. (2011). A native ChIP (NChIP) of isogenic HPT-HEK293 control cells was performed in parallel. 200 μL of eluted fractions from the first HPC4 affinity column, or 10 μL of input chromatin fractions diluted in 200 μL, were used for DNA extraction from two independent biological HPT-BAHD1 replicates or one HPT-HEK293 sample. DNA was purified by two extractions with 200 μL of phenol:chloroform:isoamylalcohol (25:24:1, pH = 8). Residual phenol was eliminated from the aqueous phase by extraction with chloroform, after which DNA was precipitated by addition of 900 μL of ethanol, 100 μL of ammonium acetate 7.5 M, and 0.2 μL of glycogen (20 μg/μL), incubation at −80°C for 30 min and centrifugation at 4°C, 20,000 × g for 15 min. The pellet was washed once in 100 μL of ethanol, then suspended in 50 μL of pure water for inputs samples, while 25 μL was used for NChIP samples.

For high-throughput sequencing, libraries were constructed by Fasteris SA (Geneva, Switzerland) from Input and NChIP samples, according to the Illumina guidelines. Independent libraries were produced for two biological replicates of the experiment, R1 and R2, input and control NChIP. The DNA colony template library was sequenced as single reads of 50 bp. Sequencing was multiplexed at three libraries per channel. We acquired a minimum of ~37 million aligned unique reads for HPT-BAHD1 and HPT-HEK293 NChIP and inputs, and used stringent cut-offs for peak calling and statistical analysis of replicate DNAs. Trimmed fastq files were mapped with *Bowtie* version 0.12.7 to the female human genome (UCSC hg19). Only uniquely mapping reads were used for further analysis; clonal reads were removed. Initial peak calling was performed with *CCAT* version 3.0 (Xu et al., 2010) with the following parameters: fragmentSize = 150, slidingWinSize = 500, movingStep = 50, isStrandSensitiveMode = 0, minCount = 4, outputNum = 10000, randomSeed = 123456, minScore = 5.0, bootstrapPass = 50. A minimum of five-fold change of NChIP over input was required for peak calling. Further analysis was performed on the regions identified by CCAT with *edgeR* (Robinson et al., 2010), by binning the genome into 200 and 400 bp non-overlapping windows. Reads were extended of 200 bp to reflect the original size of the fragment. Empty windows and outliers (>400 counts per bin) were excluded from the analysis. *edgeR* analysis was performed with a generalized linear model, excluding the effect of input and HPT-HEK293 control NChIP from the model. Full datasets have been deposited in the Gene Expression Omnibus (GEO, http://www.ncbi.nlm.nih.gov/geo) and are accessible through GEO series accession number GSE53372.

### Data annotation

In order to investigate qualitative differences between samples, data were subset for regions corresponding to genomic features of interest. Annotations from Ensembl (version 72) were retrieved using the interface *biomaRt*, which accesses *ensembl* via *BioMart*, a federated system of databases. Annotation was performed using custom scripts and functions built in the *IRanges, GenomicFeatures* and *ChIPpeakAnno*. The *UCSC* CpG island track was imported into the R environment using the package *rtracklayer*. CpG island shores were defined as those regions up to 2000 bp upstream and downstream of CpG islands. The annotation for the human genome repeat regions was downloaded from the repeat masker website hosted at the *Institute for Systems Biology* (http://www.repeatmasker.org/cgi-bin/AnnotationRequest). The lncRNA annotation (*UCSC* track: lincRNAsTranscripts) was obtained from Cabili et al. (2011).

### Gene expression microarrays

RNA quality was monitored using Agilent RNA Pico LabChips (Agilent Technologies, Palo Alto, CA). One hundred nanogram of RNA from HEK-CT or HEK-BAHD1 cells, was used as templates for the synthesis of hybridization probes for Affymetrix GeneChip Microarrays (Genechip HuGene 1.0 ST). Hybridization was carried out with three biological replicates according to the expression analysis technical manual with wash and stain kit (Affymetrix). Gene-level expression values were derived from the CEL file probe-level hybridization intensities using the model-based Robust Multichip Average algorithm (RMA; Bolstad et al., 2003). RMA performs normalization, background correction and data summarization. An analysis was performed using the LPE test (Jain et al., 2003) and a *p*-value threshold of *p* < 0.05 was used as the criterion for expression. The estimated false discovery rate (FDR) of this analysis was calculated using the Benjamini-Hochberg (BH) procedure in order to correct for multiple comparisons. Results were further subset on absolute median difference >0.2. All data is MIAME compliant and the raw data have been uploaded to the Gene Expression Omnibus database (GEO series accession number GSE51868).

### RNA extraction, reverse transcription, and quantitative PCR (RT-QPCR)

Total RNA from HEK-CT and HEK-BAHD1 cells was extracted using the RNeasy Mini Kit (Qiagen), from three biological replicates. Genomic DNA was removed by treatment with TURBO DNA-freeTM kit (Ambion). cDNAs were generated from 1 to 2 μg total RNA using the RT2-HT first strand kit (Qiagen/SABiosciences). Quantitative PCR was performed on Bio-Rad MyIQ or cfx384 devices (Biorad), using SsoFast Evagreen supermix (Biorad), as specified by the supplier, using described *BAHD1, IGF2*, and *GAPDH* primers (Bierne et al., 2009). Each reaction was performed in triplicate. Data were analyzed by the ΔΔCt method. Target gene expression data were normalized to the relative expression of the *GAPDH* reference gene.

## Results

### Generation of a human cell line stably producing BAHD1

**Figure 1:**
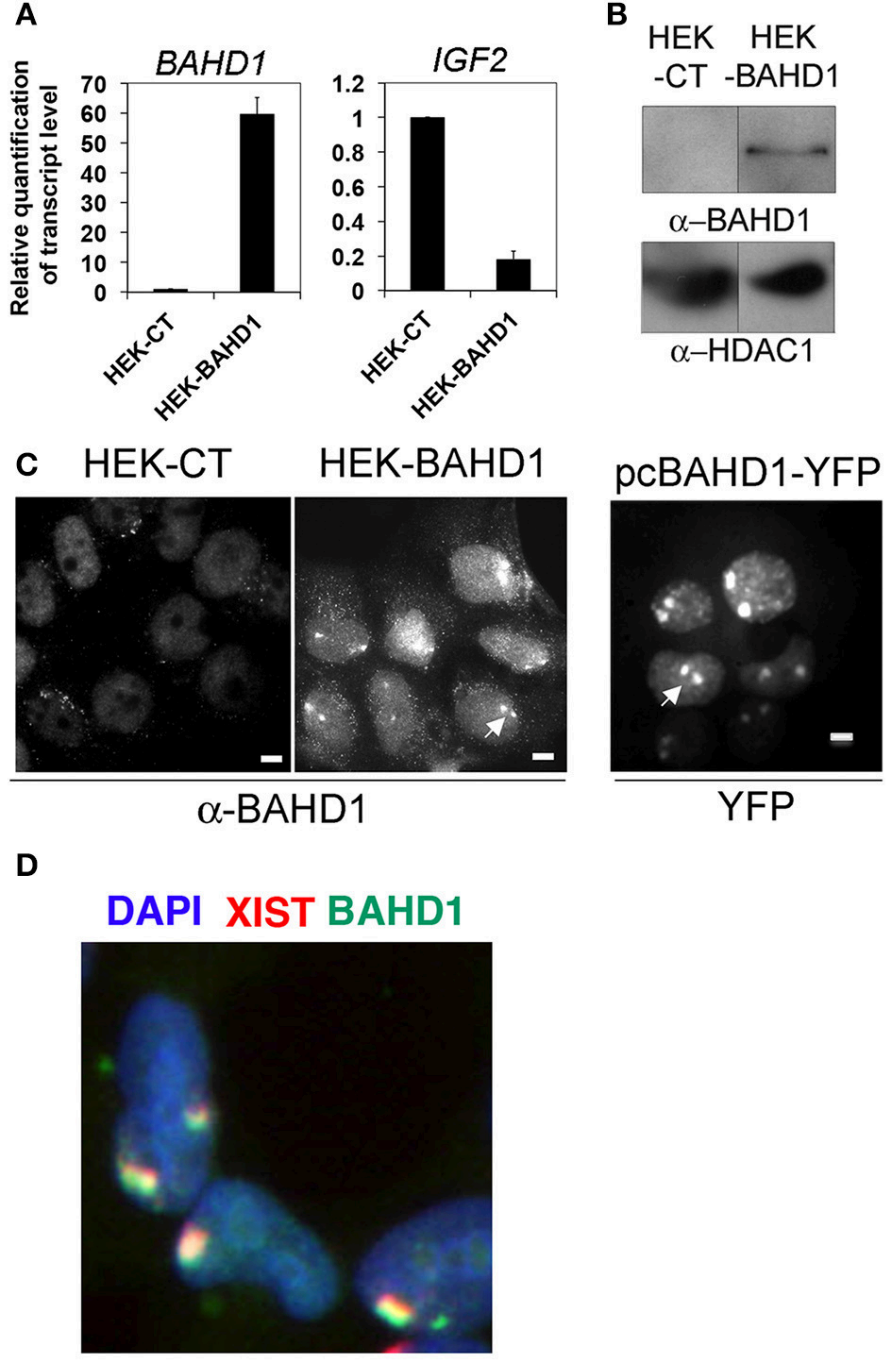
**Constitutive expression of BAHD1 in HEK-BAHD1 cells**. **(A)** RT-QPCR measurement of *BAHD1* and *IGF2* mRNA levels in HEK-BAHD1 relative to that in HEK-CT. Bars represent mean±SD of three replicates. **(B)** Immunoblot of chromatin extracts from HEK-CT and HEK-BAHD1 with BAHD1 or HDAC1 antibodies. Endogenous BAHD1 is undetectable in the control line. **(C)** Immunofluorescence studies of BAHD1 location in HEK-CT, HEK-BAHD1, or in HEK293 cells transfected with a plasmid expressing BAHD1-YFP. Scale bars, 5 μm. BAHD1 localizes to Xi in cells overexpressing BAHD1 from a chromosomal integration or from a plasmid (arrows). **(D)** Overlay image of an immunoFISH assay with anti-BAHD1 antibodies (green), combined with Xist RNA FISH (red), and staining of nuclei with DAPI (blue).

In order to investigate the effect of BAHD1 overexpression on the dynamics of DNA methylation, we generated a HEK293 cell line with stable expression of *BAHD1* (referred to as HEK-BAHD1) by integration of a single copy of the *BAHD1* coding sequence under the control of the human cytomegalovirus (CMV) promoter in the HEK293 genome. We also produced an isogenic control line (referred to as HEK-CT), by integration of the empty vector. The increase in *BAHD1* mRNA levels by ~60 fold in HEK-BAHD1 cells (Figure 1A) enabled the detection of the BAHD1 protein in HEK-BAHD1 cells, in chromatin extracts (Figure 1B), as well as in nuclei by immunofluorescence microscopy, using BAHD1 antibodies (Figure 1C). In contrast, endogenous BAHD1 was undetectable in control HEK-CT chromatin (Figure 1B), consistent with the low expression of BAHD1 in HEK293 cells and many other cell lines (Bierne et al., 2009). We have previously shown that BAHD1 represses expression of the *IGF2* gene in HEK293 cells transiently expressing *BAHD1* from a plasmid (Bierne et al., 2009). In agreement with these data, *IGF2* mRNA levels decreased by 6 fold in HEK-BAHD1 cells, when compared to control cells (Figure 1A). HEK293 are female cells with an atypical karyotype, with often two copies of the inactive X chromosome (Xi). The two Xi are visible as large heterochromatic bodies (Gilbert et al., 2000) to which BAHD1 is recruited (Bierne et al., 2009). Accordingly, we observed that BAHD1 was enriched at the Xi in HEK-BAHD1 cells, as shown by labeling of BAHD1 and XIST RNA (Figure 1D). The BAHD1 staining was less intense in HEK-BAHD1 cells than in cells transiently transfected with an YFP-BAHD1-expressing plasmid (Figure 1C). Thus, HEK-BAHD1 cells stably expressing BAHD1 can be used as a model system to study whether increasing BAHD1 cellular levels affect DNA methylation.

### *BAHD1* overexpression differentially changes the DNA methylation landscape of autosomes and chrX

**Figure 2:**
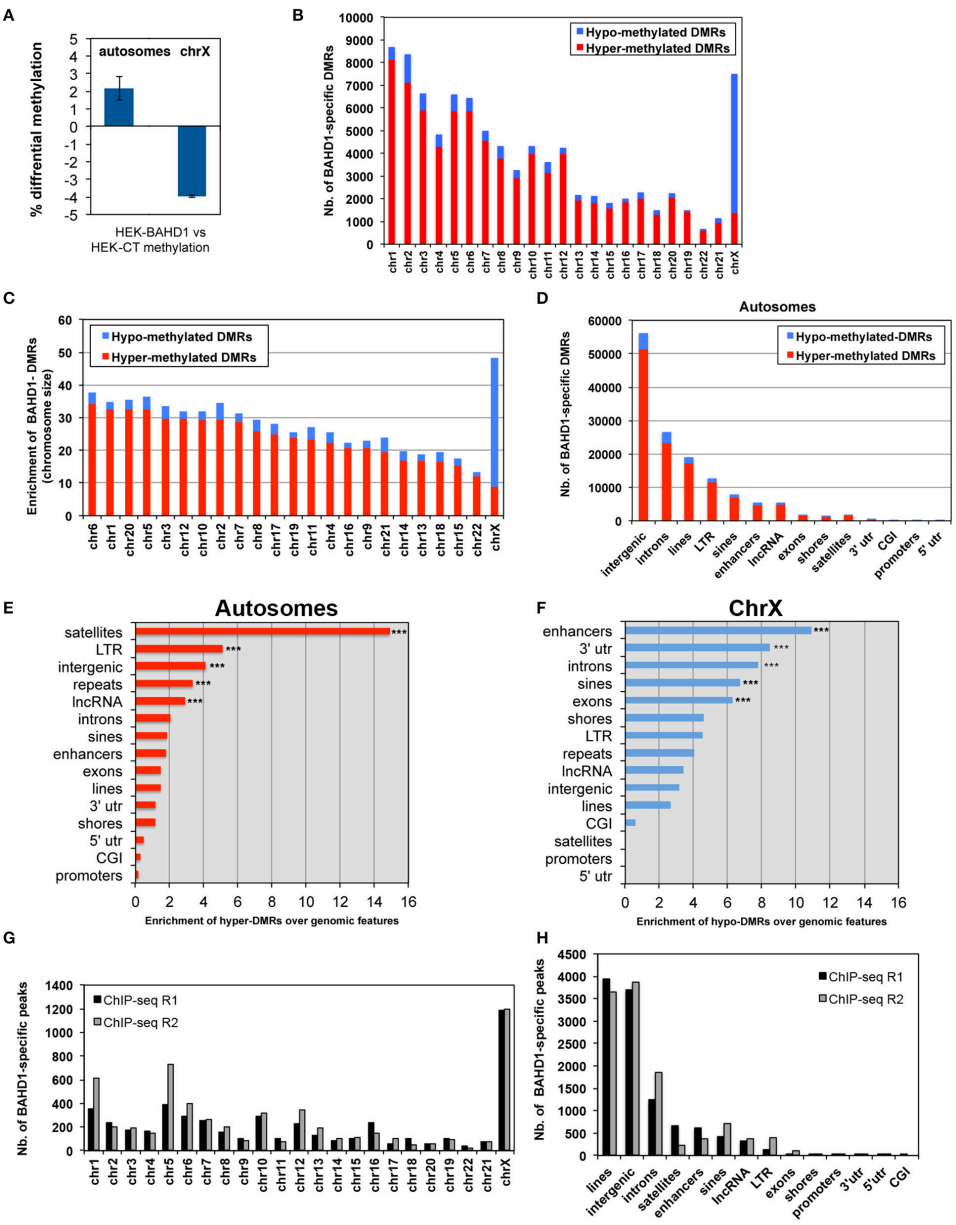
**BAHD1-induced differential methylation among chromosomes and genomic elements. (A)** Global difference in the percentage of CpG methylation level between HEK-CT and HEK-BAHD1 DNA in autosomes and chrX (methylation level = 100 ^*^ total mC reads/all reads). Results are mean±SD of two biological replicates. **(B)** Number of BAHD1-specific DMRs in autosomes and chrX of HEK-BAHD1 cells relative to that in HEK-CT cells. Hypermethylated DMRs: red. Hypomethylated DMRs: blue. **(C)** Enrichment of BAHD1-DMRs relative to autosome size; chrX is shown apart. **(D)** Number of BAHD1-DMRs in genomic elements. **(E,F)** Relative enrichment in genomic elements of BAHD1-hyper-DMRs on autosomes **(E)** or BAHD1-hypo-DMRs on chrX **(F)**. The proportion of DMRs was normalized by the length and abundance of the queried element in the genome. Regions significantly over-represented in differentially methylated regions are indicated (^***^p < 0.001). **(G,H)** Number of BAHD1-specific peaks in HPT-BAHD1 NChIP replicates in chromosomes **(G)** or genomic elements **(H)**.

In order to obtain high-resolution DNA methylation profiling, we performed whole-genome BS-seq (MethylC-seq/BS-seq Lister et al., 2009) of two HEK-BAHD1 replicates and of the HEK-CT control DNA, generating complete reference methylomes of these cell lines at single base resolution (see Materials and Methods and Supplementary Figure S1A for detailed analytical procedures). We verified that our BS-seq results were in agreement with partial methylomes previously released for HEK293 cells in ENCODE RRBS and HEK293 chromosome 21 datasets (Zhang et al., 2009; ENCODE Project Consortium, 2012; Supplementary Figure S2). Comparison of these methylomes highlighted a significant gain of methylation (on average ~2 ± 0.66%) in autosomes of HEK-BAHD1 cells, when compared to autosomes of HEK-CT cells, corresponding to methylation of ~478,000 CpG. In contrast, the average methylation level on chrX decreased by ~4 ± 0.05% (Figure 2A; Table 1). Differential analysis of HEK-CT and HEK-BAHD1 methylome replicates (Fisher's exact test, *p* < 0.1, BH-corrected, minimum methylation difference 25%) identified 91,358 regions of 300 bp that became reproducibly differentially methylated in the two biological replicates of the HEK-BAHD1 DNA, when compared to the isogenic control. 83,850 of these differentially methylated regions (herein referred to as “BAHD1-DMRs”) mapped on autosomes and 7508 on chrX (Figure 2B; Table 2). Relative to chromosome size, the highest enrichment of BAHD1-DMRs was on chrX (Figure 2C; Table 2). In agreement with global methylation levels, 89.5% of BAHD1-specific DMRs identified on autosomes showed a gain of methylation (referred to as “hyper-DMRs”), whereas 81.8% DMRs on chrX showed a loss of methylation (“hypo-DMRs”). These results indicate that up-regulation of BAHD1 induces *de novo* cytosine methylation on autosomes and loss of methylation on chrX.

**Table 1:** **Average percentage of methyl-cytosines per chromosome in HEK-CT and HEK-BAHD1 cells**.

**Chromosome**	**HEK-CT**	**HEK-BAHD1**		**HEK-BAHD1**	
		**Replicate1**	**Replicate2**	**Mean**	**sd**
chr1	**70.09**	72.06	72.47	**72.27**	0.29
chr2	**67.45**	69.2	70.32	**69.76**	0.79
chr3	**67.34**	69.01	69.75	**69.38**	0.52
chr4	**59.67**	61.31	61.68	**61.5**	0.26
chr5	**61.81**	61.66	61.48	**61.57**	0.13
chr6	**68.21**	70.46	71.4	**70.93**	0.66
chr7	**67.21**	69.13	70.23	**69.68**	0.78
chr8	**60.9**	63.21	63.85	**63.53**	0.45
chr9	**71.45**	73.1	74.42	**73.76**	0.93
chr10	**65.97**	67.84	67.33	**67.59**	0.36
chr11	**66.45**	68.06	68.92	**68.49**	0.61
chr12	**66.03**	68	68.88	**68.44**	0.62
chr13	**63.38**	65.22	65.63	**65.43**	0.29
chr14	**67.08**	68.29	69.75	**69.02**	1.03
chr15	**71.58**	73.15	74.24	**73.7**	0.77
chr16	**57.3**	57.39	60.03	**58.71**	1.87
chr17	**75.16**	76.47	77.8	**77.14**	0.94
chr18	**63.89**	65.24	66.41	**65.83**	0.83
chr19	**70.6**	71.93	73.17	**72.55**	0.88
chr20	**71.71**	73.71	75.27	**74.49**	1.1
chr21	**63.06**	67.94	67.97	**67.96**	0.02
chr22	**76.1**	77.47	79.45	**78.46**	1.4
Mean autosomes	**66.93**	68.63	69.57	**69.1**	0.66
chrX	**42.91**	38.97	38.9	**38.94**	0.05

*Bold values highlight methylation level in each chromosome in HEK-CT (one BS-seq) and HEK-BAHD1 (mean of two independent BS-seq). These data have been processed as described in Li et al. (2010)*.

**Table 2:** **Numbers of BAHD1-specific DMRs per chromosome**.

**chr**	**Total DMRs**	**Hyper-DMRs**	**% Hyper-DMRs**	**Hypo-DMRs**	**% Hypo-DMRs**	**Chromosome length (bp)**	**Nb. of DMRs relative to chromosome length**
**chrX**	**7508**	**1368**	**18.2**	**6140**	**81.8**	1.55E+08	4.84E-05
chr6	6447	5871	91.1	576	8.9	1.71E+08	3.77E-05
chr5	6587	5877	89.2	710	10.8	1.81E+08	3.64E-05
chr20	2232	2055	92.1	177	7.9	6.30E+07	3.54E-05
chr1	8676	8128	93.7	548	6.3	2.49E+08	3.48E-05
chr2	8373	7106	84.9	1267	15.1	2.43E+08	3.44E-05
chr3	6649	5902	88.8	747	11.2	1.98E+08	3.36E-05
chr10	4333	3979	91.8	354	8.2	1.36E+08	3.20E-05
chr12	4263	3988	93.5	275	6.5	1.34E+08	3.19E-05
chr7	4987	4576	91.8	411	8.2	1.59E+08	3.13E-05
chr8	4316	3783	87.7	533	12.3	1.46E+08	2.95E-05
chr17	2288	2014	88	274	12	8.12E+07	2.82E-05
chr11	3638	3157	86.8	481	13.2	1.35E+08	2.70E-05
chr19	1515	1414	93.3	101	6.7	5.91E+07	2.56E-05
chr4	4845	4287	88.5	558	11.5	1.91E+08	2.54E-05
chr21	1152	942	81.8	210	18.2	4.81E+07	2.39E-05
chr9	3259	2907	89.2	352	10.8	1.41E+08	2.31E-05
chr16	2019	1865	92.4	154	7.6	9.04E+07	2.24E-05
chr14	2121	1803	85	318	15	1.07E+08	1.98E-05
chr18	1514	1301	85.9	213	14.1	7.81E+07	1.94E-05
chr13	2151	1929	89.7	222	10.3	1.15E+08	1.87E-05
chr15	1807	1559	86.3	248	13.7	1.03E+08	1.76E-05
chr22	678	613	90.4	65	9.6	5.13E+07	1.32E-05
**Total autosomes**	**83850**	**75056**	**89.51**	**8794**	**10.49**		

*This table includes counts for non-overlapping 300 bps windows, which have been found differentially methylated in HEK-BAHD1, when compared to HEK-CT BS-seq, and which localize to individual chromosomes. DMRs are divided in hyper-methylated (more methylation than control: hyper-DMRs) and hypo-methylated (less methylation than control: hypo-DMRs). The results are ranked according to the enrichment of DMRs relative to the chromosome size. Bold Letters highlight values in chrX and in all autosomes*.

### Distribution of BAHD1-specific DMRs on autosomes

We next examined the location of BAHD1-DMRs in different genomic elements on autosomes. The majority of DMRs were principally outside of CpG islands and mapped on intergenic regions, introns and interspersed repeats (LINEs, LTR, SINEs; Figure 2D). Relative to the size of each genomic element, hyper-DMRs were particularly enriched at satellites and other repeat sequences, intergenic regions and lncRNAs, whereas they rarely mapped in 5′UTRs, CGIs, and promoters (Figure 2E; Table 3A). Subsets of hypermethylated BAHD1-DMRs were observed at enhancers (4579) and CGI-shores (1175), which like CGIs, are cis-regulatory modules that play important role in regulation of gene expression (Schilling and Rehli, 2007; Irizarry et al., 2009). The % of mCpG can be divided into low levels (0–25%), “partially methylated domains” (PMD) and high levels (75–100%; Lister et al., 2009; Hon et al., 2012). The directional change in methylation in response to overexpression of BAHD1 went toward a higher methylation (Table 4A).

**Table 3:** **Number and relative enrichment of BAHD1-specific DMRs in genomic elements in autosomes and X chromosome**.

**Genomic elements**	**Hyper-DMRs autosomes**	**% Hyper-DMRs on all autosomal DMRs**	**Enrichment**	**Genomic elements**	**Hypo-DMRs autosomes**	**% Hypo-DMRs on all autosomal DMRs**	**Enrichment**
**A. AUTOSOMES**							
Satellites	1446	99.1	**14.9**	LTR	1151	9.1	**0.5**
LTR	11560	90.9	**5.1**	Enhancers	1005	18	**0.4**
Intergenic	51242	91	**4.1**	Repeats	4397	9.7	**0.4**
Repeats	40871	90.3	**3.4**	Intergenic	5086	9	**0.4**
lncRNA	4935	90.3	**2.9**	CGI	72	55.4	**0.3**
Introns	23132	86.6	**2.1**	Shores	299	20.3	**0.3**
Sines	6920	88.4	**1.8**	3′-UTR	78	18.8	**0.3**
Enhancers	4579	82	**1.8**	Exons	285	14.8	**0.3**
Lines	17105	90.2	**1.5**	Introns	3568	13.4	**0.3**
Exons	1646	85.2	**1.5**	lncRNA	529	9.7	**0.3**
3′-UTR	337	81.2	**1.2**	Sines	904	11.6	**0.2**
Shores	1175	79.7	**1.2**	Lines	1848	9.8	**0.2**
5′-UTR	34	79.1	**0.5**	5′-UTR	9	20.9	**0.1**
CGI	58	44.6	**0.3**	Satellites	13	0.9	**0.1**
Promoters	76	89.4	**0.2**	Promoters	9	10.6	**0**
**Genomic elements**	**Hyper-DMRs chrX**	**% Hyper-DMRs on all chrX DMRs**	**Enrichment**	**Genomic elements**	**Hypo-DMRs chrX**	**% Hypo-DMRs on all chrX DMRs**	**Enrichment**
**B. X CHROMOSOME**							
Satellites	280	100	**38.7**	Enhancers	1179	91.5	**10.9**
Shores	39	22	**1.3**	3′-UTR	87	89.7	**8.5**
Intergenic	970	26.9	**1.2**	Introns	3284	89.9	**7.8**
Repeats	942	22.8	**1.2**	Sines	1024	88.8	**6.7**
3′-UTR	10	10.3	**1**	Exons	248	89.9	**6.3**
Enhancers	109	8.5	**1**	Shores	138	78	**4.6**
LTR	141	17.3	**0.9**	LTR	672	82.7	**4.5**
Introns	367	10.1	**0.9**	Repeats	3193	77.2	**4.1**
Lines	318	22.1	**0.8**	lncRNA	191	88	**3.4**
Sines	129	11.2	**0.8**	Intergenic	2634	73.1	**3.1**
Exons	28	10.1	**0.7**	Lines	1123	77.9	**2.7**
lncRNA	26	12	**0.5**	CGI	4	100	**0.6**
5′-UTR	0	0	**0**	5′-UTR	0	0	**0**
CGI	0	0	**0**	Promoters	0	0	**0**
Promoters	0	0	**0**	Satellites	0	0	**0**

*This table includes counts for non-overlapping 300 bps windows, which have been found differentially methylated in HEK-BAHD1, when compared to HEK-CT BS-seq, and which localize to individual genomic elements. DMRs are divided in hyper-methylated (more methylation than control, “hyper-DMRs”) and hypo-methylated (less methylation than control, “hypo-DMRs”). The results are ranked according to the enrichment relative to the size of each genomic feature, as highlighted in bold*.

**Table 4:** **Average difference in global methylation levels per genomic elements between HEK-CT and HEK-BAHD1 cells**.

	**Average Difference between HEK-BAHD1 and HEK-CT**		
	**Low (0–25%)**	**PMD (25–75%)**	**High (75–100%)**
**A. AUTOSOMES**			
Satellite	−6.1	−1.52	7.61
LTR	−1.85	−4.21	6.06
Intergenic	−1.6	−4.25	5.85
lncRNA	−1.47	−4.35	5.82
Lines	−2.07	−3.18	5.24
Enhancers	−0.13	−3.26	3.38
Introns	−0.43	−2.88	3.29
Sines	−0.61	−2.41	3.01
Shores	0.04	−2.6	2.54
Exons	0.25	−2.02	1.78
Promoters	0.27	−1.9	1.62
3′-UTR	−0.1	−0.62	0.7
CGI	0.53	−1.06	0.52
5′-UTR	0.68	−0.94	0.25
**B. chrX**			
Satellite	−9.46	−10.96	20.68
LTR	0.47	5.31	−5.8
Intergenic	0.2	4.54	−4.76
lncRNA	2.85	4.24	−7.09
Lines	0.31	2.79	−3.11
Enhancers	−1.11	15.11	−14.01
Introns	0.51	11.9	−12.41
Sines	0.34	12.09	−12.43
Shores	−4.94	14.8	−9.86
Exons	−2.59	12.54	−9.96
Promoters	−7.27	13.69	−6.43
3′-UTR	1.15	13.42	−14.57
CGI	−10.41	12.21	−1.81
5′-UTR	−10.71	12.93	−2.22

*The methylation level ranges were defined into low levels (0–25%), partially methylated domains (25–75%), and high levels (>75%). The average difference indicates subtraction of HEK-BAHD1 (replicates 1 and 2) from HEK-CT methylation levels, with positive and negative differences in red and blue boxes, respectively*.

We searched for the presence of binding sites for BAHD1-associated partners in hypermethylated BAHD1-DMRs on autosomes by examining the overlap of DMRs and binding sites for transcription regulators listed in the ENCODE TFBS (Transcription Factor Binding Sites) cluster track. Remarkably, the two first transcription regulators of this analysis (Supplementary Table S1) were SETDB1 and KAP1, two known partners of BAHD1. HP1-γ, HDAC2 and the transcription factor SP1, which also co-precipitate with BAHD1 in HEK293 cells (Bierne et al., 2009; Lebreton et al., 2011) were also in this list. Additionally, there were binding sites for EZH2, the H3K27 KMT that induces H3K27me3, a mark associated with BAHD1 heterochromatic foci (Bierne et al., 2009), and STAT transcription factors (STAT1, STAT3, and STAT5A) that can be functionally related to BAHD1-mediated repression of interferon-stimulated genes (Lebreton et al., 2011). These findings support the hypothesis that BAHD1, in association with its partners within macromolecular complexes, is mechanistically linked to the establishment of DNA methylation patterns on autosomes.

### Distribution of BAHD1-specific DMRs on chrX

In contrast to what was observed on autosomes, comparison of methylation profiles on chrX in HEK-CT and HEK-BAHD1 DNA revealed a puzzling loss of methylation on all genomic elements, from the high level to partially methylated level (Table 4B), with the exception of satellites that gained methylation like in autosomes. DMR analysis emphasized unique patterns of differential methylation on chrX in BAHD1-overexpressing cells. Relative to the size of each genomic element, DMR counts at enhancers, gene bodies (3′-UTR, introns, exons) and SINEs showed the highest pattern of hypomethylation on chrX (Figure 2F). There were only 4 BAHD1-DMRs on CGIs (all hypo-methylated) and no DMRs on 5′-UTR and promoters (Table 3B). Thus, in contrast to autosomes, most BAHD1-DMRs on chrX are enriched in enhancers and gene bodies and are hypo-methylated in comparison with the same regions in control cells.

### Relationship between BAHD1-associated differential DNA methylation and gene expression

**Figure 3:**
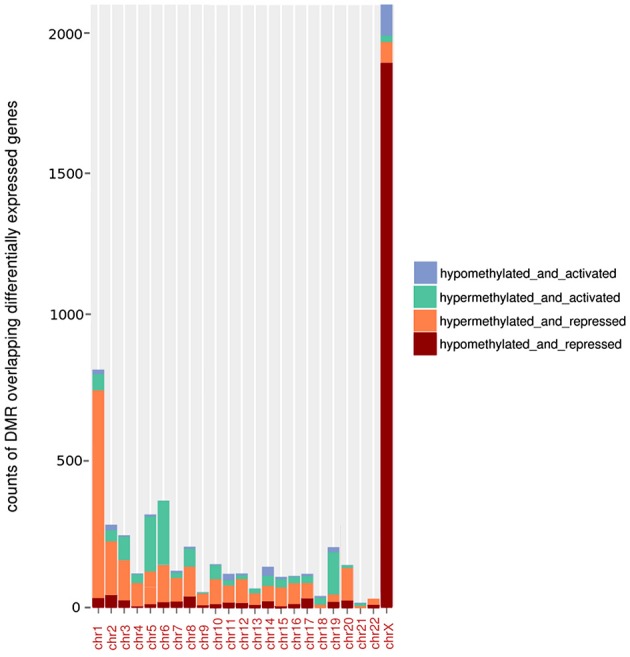
**Relationship between BAHD1-DMRs and gene expression**. Histograms represent, for each autosome and chrX, the number (“counts”) of hypermethylated or hypomethylated DMRs that are located within a window of 10 kb upstream to 10 kb downstream of a gene differentially expressed (activated or repressed) in HEK-BAHD1 cells compared to HEK-CT.

With the aim of investigating the potential association between BAHD1-induced DNA methylation changes and gene expression, we generated transcriptome datasets of HEK-CT and HEK-BAHD1 lines, using Affymetrix DNA arrays. Analysis of differential gene expression between the two lines yielded 1304 up-regulated and 1137 down-regulated transcripts upon BAHD1 overexpression (LPE, BH-adjusted *p* < 0.05). Given the role of BAHD1 in transcriptional repression, our analysis principally focused on genes that were down-regulated in HEK-BAHD1 cells. We found BAHD1-specific DMRs in 701 (61%) repressed genes, within a window encompassing 10 kb upstream to 10 kb downstream of each gene. In most autosomes, there was a significantly higher proportion of hyper-DMRs than hypo-DMRs associated with repressed genes (Figure 3), suggesting that BAHD1-associated *de novo* DNA methylation on autosomes is mainly associated with gene repression.

On chrX, analysis of the transcriptome datasets identified 119 genes that were differentially expressed in HEK-BAHD1 when compared to HEK-CT cells, of which 72 (60%) were down-regulated. In contrast to autosomes, the majority of chrX genes that were repressed in HEK-BAHD1 cells were associated with hypomethylated DMRs in both BS-seq replicates (i.e., 58/72; Supplementary Table S2; Figure 3). Since BAHD1 targets the Xi (Figure 1), we searched for evidence linking BAHD1-associated repression to known methylation changes on the Xi. As mentioned above, there was no BAHD1-specific hyper-DMR on CGIs of chrX (Table 3B), ruling out an effect of BAHD1 in CGI methylation on the Xi. We investigated whether chrX genes that were down-regulated in HEK-BAHD1 were known to be always inactivated on Xi, or could escape X inactivation, by using published data on the status of Xi genes (Zhang et al., 2013). Thirty-two repressed genes were present in this list, of which 13 were classified as “always inactive” and 19 as “heterogeneous” (Supplementary Table S2). Taken together, these results suggest that repression of a set of chrX genes in HEK293 cells overexpressing BAHD1 might be due to loss of methylation on Xi, opening the possibility that BAHD1 might be involved in heterogeneous repression on Xi-linked genes.

### BAHD1-specific DMRs are clustered into large chromosomal domains

**Figure 4:**
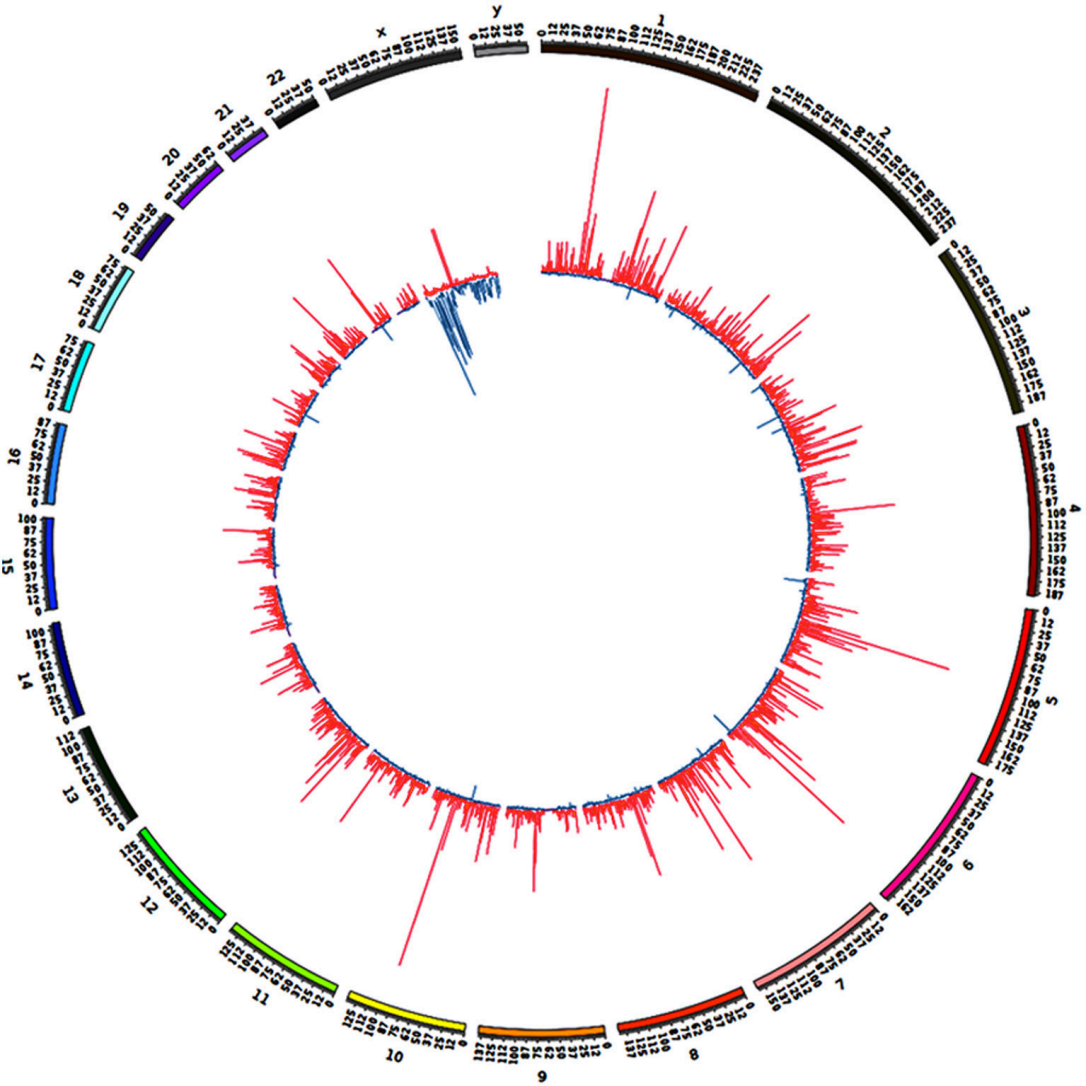
**Distribution of BAHD1-DMR clusters across the genome**. DMRs present in HEK-BAHD1 cells in comparison to HEK-CT cells were binned into 0.5 Mb windows as “BAHD1-DMR clusters” represented by bars. The distribution of clusters across the genome of HEK-BAHD1 cells, with hypermethylated clusters in red and hypomethylated clusters in blue, is plotted with Circos (Krzywinski et al., 2009).

**Figure 5:**
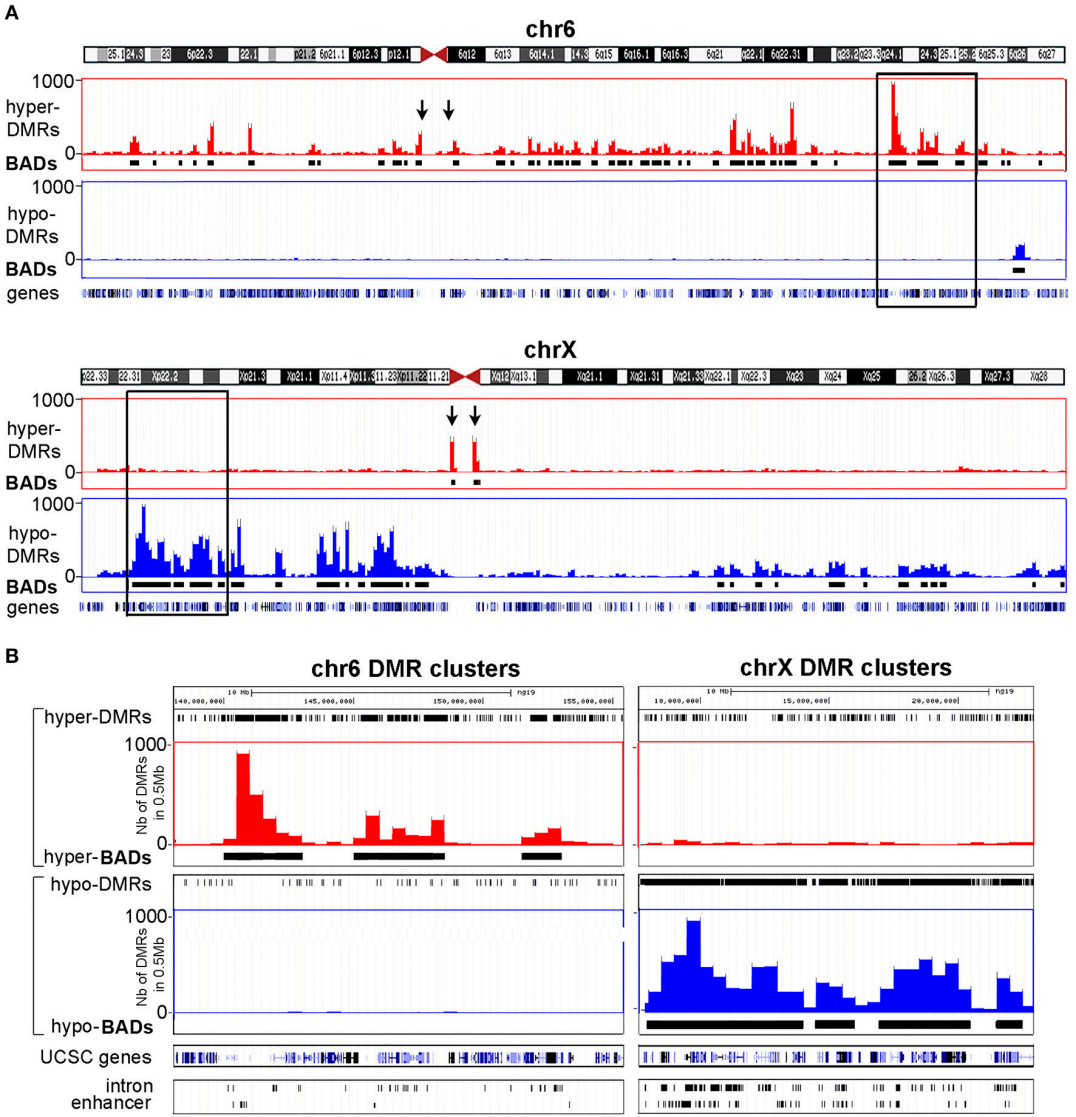
**BAHD1-specific domains (BADs)**. Large-scale genomic organization of DMRs present in HEK-BAHD1 cells in comparison to HEK-CT cells is illustrated for an autosome (chr6) and for chrX. Scale indicates the number of DMRs per 0.5 Mb window. Bars represent clusters of DMRs with gain of methylation (hyper-DMRs, in red) or loss of methylation (hypo-DMRs, in blue). BADs (indicated by black boxes) correspond to contiguous domains of DMR clusters where DMR counts are in the top quartile of the genome-wide DMR distribution. The position of genes is shown below. **(A)** Chromosome-wide representation of hypermethylation and hypomethylation domains on chr6 (top) and chrX (bottom). Arrows indicate the position of pericentromeric regions. **(B)** Magnification of the domain squared in A, with a chr6 hypermethylation domain (left) and a chrX hypomethylation domain (right). The position of DMRs in introns and enhancers is shown below.

We previously noticed that high-level overexpression of *BAHD1* from a plasmid in HEK293 cells triggers massive compaction of chromatin visible when observed in electron microscopy (Bierne et al., 2009). We hypothesized that BAHD1-mediated heterochromatinization might spread and that this event might coincide with BAHD1-associated DNA methylation changes on large regions. To address this hypothesis, the overall genomic distribution of BAHD1-DMRs was examined at a higher scale by binning DMRs into 0.5 Mb windows (i.e., “BAHD1-DMR clusters”). This analysis revealed that BAHD1-DMRs were non-uniformly distributed along the whole human genome (Figure 4). In order to refine the mapping of regions with high density of DMRs, we defined contiguous domains in DMR clusters where the DMR counts were in the top quartile of the genome-wide DMR distribution. Examples of such “BAHD1–Associated differentially methylated Domains” (BADs) are shown in Figure 5, for chr6 and chrX. Overall, we found 839 BADs with sizes of 0.3–6.5 Mb (median 0.5 Mb), mostly hypermethylated in autosomes (792 “hyper-BADs”) and hypomethylated in chrX (26 “hypo-BADs”).

Several genome-wide analyses have revealed diverse chromatin domains that play important roles in nuclear organization and function (Hu et al., 2012; Padeken and Heun, 2014). Of these, Lamina-Associated Domains (LADs) are proposed to be dynamic heterochromatic structures located to the nuclear periphery and correlated with gene repression (Guelen et al., 2008; Luperchio et al., 2014). Interestingly, in autosomes, 60% of hyper-BADs overlapped with LADs (*p* < 0.001, hyperG test). These results highlight the possibility that BAHD1-induced DNA methylation is linked to *de novo* formation of heterochromatic domains at the nuclear periphery. In addition, we noticed that in agreement with the hypermethylation of satellites found in our pattern analyses (Figure 2; Table 3), a set of hyper-BADs, including those located on chrX, mapped to pericentromeric regions (Figure 5), which are known regions of constitutive heterochromatin. Thus, BAHD1-associated chromatin repressive complexes might play a role in heterochromatinization of pericentromeric satellites by increasing their 5 mC content. The repartition of hypo-BADs on chrX was also non-random. Hypo-BADs particularly mapped in enhancers and gene bodies (introns, exons) and were more abundant on the short arm of chrX (Xp) than on the long arm (Xq), and absent from the central region, in particular on the proximal part of Xq (Figure 5). Overall, these results suggest that overexpression of BAHD1 may dynamically alter heterochromatin topology by formation of large differentially methylated domains with distinct consequences on chrX and autosomes.

### Mapping of BAHD1-occupancy domains in response to *BAHD1* overexpression

We next aimed to find out whether overexpression of BAHD1 stimulates binding of BAHD1 on large chromatin domains. So far, our efforts to immunoprecipitate the endogenous BAHD1 protein using different commercial or custom antibodies has been unsuccessful. To map the genome-wide distribution of BAHD1 binding sites in response to transient induction of *BAHD1* expression, we used a HEK293 cell line stably expressing tetracycline-inducible His6-Protein C (HPC)-tagged BAHD1 (referred to as HPT-BAHD1 cells) that we generated earlier (Lebreton et al., 2011). The genomic DNA present in chromatin extracts from HPT-BAHD1 cells before (input) and after immunoprecipitation (ChIP) of the HPC-BAHD1 protein with Protein C antibodies was analyzed by high-throughput sequencing. This Native ChIP-seq (NChIP-seq) enabled the analysis of the DNA to which BAHD1 binds without any requirement for formaldehyde crosslinking. To filter out non-specific DNA binding on the affinity matrix, we also performed NChIP-seq analysis of HPT-HEK293 control chromatin extracts. A stringent analysis yielded 4936 and 5742 BAHD1-binding events (“BAHD1-peaks”) across the genome in replicates 1 and 2, respectively. Notably, the distribution of BAHD1-peaks across chromosomes showed enrichment of BAHD1 binding sites on chrX, with 24% (replicate 1) and 21% (replicate 2) of all BAHD1-peaks mapping on this chromosome (Figure 2G). This result is in agreement with enrichment of BAHD1-DMRs on chrX and with the recruitment of BAHD1 to the Xi. Regarding the distribution on genomic elements, 80% of BAHD1-peaks preferentially mapped at interspersed repeats LINEs and SINEs (37.4 ± 5.9%), intergenic regions (33.2 ± 0.1%), and introns (13.6 ± 3.4%; Figure 2G). Relative to the size of each genomic element, BAHD1-peaks were, like BAHD1-DMRs, mostly enriched at satellites (Table 5).

**Table 5:** **Number and relative enrichment of BAHD1-specific peaks in genomic elements in HPT-BAHD1 NChIP-seq replicates**.

**Genomic element**	**Nb. of BAHD1 peaks Replicate 1**	**Nb. of BAHD1 peaks Replicate 2**	**Replicate 1 enrichment**	**Replicate 2 enrichment**	**Mean enrichment**
Satellites	675	234	2.183	0.757	**1.47**
Lines	3940	3647	0.151	0.14	**0.1455**
Intergenic	3691	3859	0.087	0.09	**0.0885**
lncRNA	326	369	0.064	0.072	**0.068**
Enhancers	613	382	0.08	0.05	**0.065**
Introns	1240	1864	0.038	0.058	**0.048**
Sines	416	724	0.019	0.032	**0.0255**
LTR	131	391	0.011	0.034	**0.0225**
Promoters	11	24	0.009	0.02	**0.0145**
Exons	34	116	0.006	0.02	**0.013**
3′-UTR	4	20	0.004	0.018	**0.011**
Shores	13	31	0.004	0.011	**0.0075**
5′-UTR	1	5	0.002	0.011	**0.0065**
CGI	1	0	0.001	0	**0.0005**

*Mean of enrichement in biological replicates is highlighted in bold*.

**Figure 6:**
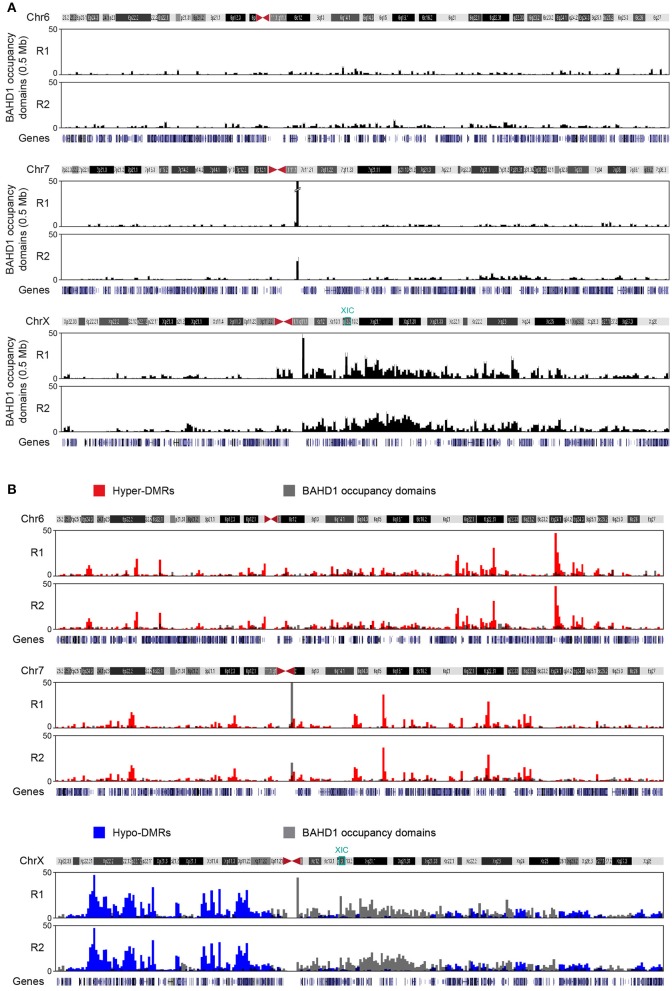
**BAHD1-specific binding domains. (A)** Large-scale genomic organization of BAHD1-binding sites (“occupancy domains”) upon induction of *BAHD1* expression for 30 h in HPT-BAHD1 cells. Sequencing DNA from NChIP of Protein-C-tagged BAHD1 generated peaks that were clustered in 0.5 Mb windows (black bars). Results are shown for two autosomes (chr6, chr7) and chrX. Scale indicates the number of peaks per bar. **(B)** Overlap of BAHD1-DMR clusters and BAHD1-occupancy domains on chr6 (hypermethylated DMRs) and chrX (hypomethylated DMRs). The position of XIC and genes is indicated.

Binning BAHD1-specific peaks into 0.5 Mb windows revealed that they clustered into larger regions, as BAHD1-DMRs. Examples of such “BAHD1–occupancy domains” are shown for chr6, chr7, and chrX on Figure 6A. Surprisingly, overlapping BAHD1–occupancy and–differentially methylated regions revealed a marked difference between autosomes and chrX. On autosomes, BAHD1-binding and–hypermethylated domains often overlapped or were adjacent. In contrast, on chrX, there was an inverse correlation between the location of BAHD1-binding and hypomethylated domains (Figure 6B). Furthermore, BAHD1-occupancy domains were more abundant on the long arm (Xq) than the short arm of chrX (Xp), particularly in the half part of Xq containing the X-inactivation center (XIC) on the Xi. Taken together, these data are in agreement with the hypothesis that BAHD1 plays distinct roles on chrX and autosomes and support a model in which BAHD1-mediated chromatin compaction coincides with binding of BAHD1 on large genomic regions.

## Discussion

In this study, we report that increasing BAHD1 cellular levels in human embryonic 293 cells is sufficient to trigger profound changes in CpG methylation patterns at both the local (kb) and large (Mb) genome scale. In addition, BAHD1-associated methylation signatures are distinct depending on location, with gain of methylation on autosomes and loss of methylation on chrX. We cannot exclude that these changes are indirect consequences of BAHD1 expression at non-physiologically high levels. Yet, our experimental data provide insight into potential functions of BAHD1 in epigenetic regulation. We hypothesize a role for BAHD1 in the modulation of DNA methylation patterning and in the spatial organization of silent chromatin.

Our results indicate that BAHD1-specific hypermethylated DMRs are enriched in binding sites for the known BAHD1-associated partners SETDB1, KAP1, HP1-γ and HDAC2, as well as for the H3K27me3 writer EZH2, which are all known to interact with *de novo* DNMTs (Fuks et al., 2001, 2003; Lehnertz et al., 2003; Li et al., 2006; Vire et al., 2006; Rush et al., 2009). Also of interest is the presence in a subset of BAHD1-DMRs of binding sites for the transcription factor SP1, with which BAHD1 co-immunoprecipitates (Bierne et al., 2009), as well as for STAT factors that may act in synergy with BAHD1 to repress immunity gene expression during a bacterial infection (Lebreton et al., 2011). In addition, the comparison of HEK293 and HEK-BAHD1 transcriptomes suggests a relationship between the location of BAHD1-DMRs and BAHD1-associated repressed genes. It is worth mentioning that BAHD1 has similarities to the metastasis-associated proteins (MTAs) of the Nucleosome Remodeling and Deacetylation (NuRD) co-repressor complex (Lai and Wade, 2011), which has been shown to couple histone deacetylation and DNA methylation (Morey et al., 2008; Latos et al., 2012). Like MTAs, BAHD1 is a BAH domain-containing protein that binds a reader of 5-methylcytosines (i.e., MBD1, Bierne et al., 2009). BAHD1 might thus act as a scaffold protein that bridges MBD1 and HDAC1/2, as MTAs, and also connects HP1 and KMTs, which can recruit DNMTs. These data converge to a model in which BAHD1-associated multiprotein complexes act in synergy with the DNA methylation machinery to form repressive chromatin.

When transiently overexpressed from plasmid-based expression systems, BAHD1 promotes the formation of nuclear heterochromatic foci (Bierne et al., 2009). These foci seem to correspond to different heterochromatin subtypes, as they are enriched with HP1-α, a marker of constitutive heterochromatin, or H3K27me3, a feature of facultative heterochromatin, but HP1-α and H3K27me3 do not strictly overlap in these foci (Bierne et al., 2009). BAHD1 is also recruited to Xi, a paradigm of facultative heterochromatin. In addition, BAHD1 interacts with chromatin regulators that play a role in the formation of both constitutive and facultative heterochromatin (i.e., HP1 proteins α, β and γ, KAP1, HDAC1/2, SETDB1). Here we show that stable expression of *BAHD1* in HEK293 cells induces *de novo* DNA methylation at different genomic elements in autosomes, predominantly at satellites, the major component of the pericentromeric heterochromatin, and other regions prone to being packaged into heterochromatin (i.e., interspersed repeats, intergenic regions, introns and sequences transcribed in non-coding RNAs). BAHD1 overexpression also increases DNA methylation at a set of cis-regulatory modules, including enhancers and CGI shores. Several studies suggest that methylation of these modules may play a more significant role in the regulation of gene expression than CGIs (Irizarry et al., 2009; Hon et al., 2013; Long et al., 2013; Ziller et al., 2013). We hypothesize that BAHD1-associated chromatin repressive complexes may recruit DNMTs to non-coding DNA repeats, thus contributing to the stabilization of constitutive heterochromatin, and/or to cis-regulatory modules, and thereby compacting these key regulatory elements into facultative heterochromatic domains leading to gene silencing. However, it is also possible that upon BAHD1 overexpression, BAHD1-interacting proteins are delocalized from various endogenous complexes, which could indirectly alter the DNA methylome.

NChIP-seq analysis of BAHD1-binding sites suggests that BAHD1 spreads on chromatin in BAHD1-overexpressing cells (Figure 6). This spreading might coincide with propagation of DNA methylation, as we identified regions with high densities of BAHD1-DMRs. Indeed, BAHD1-DMRs are non-uniformly distributed along the genome and group into large chromosomal domains, termed BADs. We find that 60% of BADs coincide with LADs, which are large genomic segments anchored at the nuclear periphery. LADs are proposed to be nuclear compartments of silent chromatin formed in response to development stimuli (Guelen et al., 2008; Luperchio et al., 2014; Padeken and Heun, 2014). The size distribution (0.1–10 Mb) and the median sequence length (0.5–0.8 Mb) of LADs are very similar to that of BADs (0.3–6.5 Mb; median 0.5 Mb). Furthermore, our previous electron microscopy experiments highlighted BAHD1-induced heterochromatin at the nuclear periphery (Bierne et al., 2009). From this, we propose that BAHD1 may mediate chromatin reorganization into *de novo* heterochromatic regions through DNA methylation changes, thereby contributing to the three-dimensional organization of chromatin within the nucleus, in response to developmental, physiological or environmental stimuli. Interestingly, a recent study compiling data from a 100 human epigenomes highlights the dynamics of mCpG distribution across tissue and cell types and emphasizes the importance of examining this dynamics at the megabase-scale (Roadmap Epigenomics et al., 2015). Taken together, these results call for future research to investigate whether BADs are formed in response to activation of the *BAHD1* endogenous gene by external stimuli. Such stimuli, and their effect on *BAHD1* expression level in different tissues, are presently unknown. It is therefore, necessary to characterize the cellular signaling pathways that control *BAHD1* expression and association with other proteins.

In this work, we also show that in HEK293 female cells overexpressing BAHD1, chrX undergoes a significant loss of DNA methylation, while being a binding hotspot for BAHD1. We cannot assign these methylation changes to specific Xi or Xa territories because the data were derived from mixed cell and chromosome populations. However, since BAHD1 is recruited to Xi, as shown by microscopy, it is tempting to speculate that overexpression of BAHD1 triggers a loss of DNA methylation on the Xi. This observation must be put into perspective within the specific context of the differential methylation of Xi compared to Xa chromosomes in female cells. Different pieces of evidence suggests that DNA methylation patterns are inverted on Xi and Xa, with CGIs and gene bodies being more and less methylated, respectively (Viegas-Pequignot et al., 1988; Prantera and Ferraro, 1990; Weber et al., 2005; Hellman and Chess, 2007). Up to now, a mechanistic explanation of the role of gene body methylation and gene silencing on Xi is still missing. We found that BAHD1-specific DMRs on chrX are clustered into hypomethylated domains on gene bodies, particularly on the short arm of chrX (Xp), in contrast with BAHD1-occupancy domains, which are abundant on the long arm (Xq). This puzzling observation opens the possibility, among other hypotheses, that overexpressed BAHD1 may sequester DNMTs at certain sites of Xq, altering X-gene body methylation *in cis* on Xp. Interestingly, two recent studies point to a possible role for BAHD1 at Xi: BAHD1 co-purifies with CDYL, a new partner of Xi (Escamilla-Del-Arenal et al., 2013) and BAHD1 interactors MBD1 and SETDB1 were found to contribute to the maintenance of X inactivation in somatic cells (Minkovsky et al., 2014). Overall, this work establishes a basis for future studies aimed at exploring the role of BAHD1 in transcription/maintenance/stability of the Xi.

## Conclusions

*De novo* changes in DNA methylation patterns induced by the stable overexpression of BAHD1 in HEK293 cells suggest that BAHD1 may play a role in the formation of distinct types of heterochromatic domains by controlling the setting and distribution of mCpG. We did not demonstrate that the DNA methylation changes observed in this study are a direct consequence of BAHD1 binding to the chromatin. To confirm these hypotheses, it is now essential to study whether the DNA methylome is remodeled upon inactivation of the endogenous *BAHD1* gene. The use of novel techniques to generate knockout cells or animals, such as the CRISPR/Cas9 technology, should facilitate the achievement of these goals, opening an exciting new area of future research on this epigenetic regulator.

## Author contributions

EL analysed the BS-seq, ChIP-seq, and transcriptome data and wrote the manuscript. AL participated in sample collections and analyzed and drafted the ChIP-seq data. GL generated the cellular model and participated in sample collections and QPCR. MD and JC participated in data analysis and drafted the BS-seq data. SB and PC participated in data analysis and obtained funding. HB conceived the study, analyzed the BS-seq, ChIP-seq, and transcriptome data, obtained funding and wrote the manuscript. All authors read and approved the final manuscript.

## Funding

This work was supported by the French National Research Agency (ANR 11 BSV3 003 01, EPILIS), the French Ligue Nationale Contre le Cancer (comité régional d'Ile-de-France, LNCC RS10/75–76 and LNCC 131/12), the European Research Council (ERC advanced grant to P. Cossart: BacCellEpi grant 670823), and Institut National de la Recherche Agronomique (AO blanc 2011, MICA). The Beck lab was supported by the Wellcome Trust (WT99148), Royal Society Wolfson Research Merit Award (WM100023) and EU-FP7 projects EPIGENESYS (257082), EpiTrain (316768), and BLUEPRINT (282510).

### Conflict of interest statement

The authors declare that the research was conducted in the absence of any commercial or financial relationships that could be construed as a potential conflict of interest.
