# Supplementary material for: Overexpression of the Heterochromatinization Factor BAHD1 in HEK293 Cells Differentially Reshapes the DNA Methylome on Autosomes and X Chromosome
Source: Front Genet. 2015 Dec 1;6:339. doi: 10.3389/fgene.2015.00339 (PMC4664705; doi:10.3389/fgene.2015.00339)
Supplement: Supplementary file 1 [file Presentation1.PDF]

Supplementary Material

Overexpression of the heterochromatinization factor BAHD1 in HEK293 cells differentially reshapes the DNA methylome on autosomes and X chromosome.

Emanuele Libertini [1,2], Alice Lebreton[3,4,5,$] , Goran Lakisic [3,4,5,6,7], Marie-Agnes Dillies [1], Stephan Beck [2], Jean-Yves Coppée [1], Pascale Cossart [3,4,5] and Hélène Bierne [3,4,5,6,7] *

*** Correspondence:** Hélène Bierne: helene.bierne@jouy.inra.fr

1 Supplementary Data

Generation of the HEK-BAHD1 and HEK-CT lines.

The BAHD1 over-expressing cell line (HEK-BAHD1) and its corresponding control line (HEK-CT) were generated as followed: full-length human BAHD1 cDNA (coding for 1-780 amino acid residues) was inserted in plasmid pcDNA5/FRT-HPT-blue, a derivative of pCDNA5/FRT/V5-His from Invitrogen, carrying the N-terminal Tag His-PC instead of V5-His (Lebreton et al., 2011), in FseI et AscI restriction sites, leading to pcDNA5/FRT-HPT-BAHD1. This plasmid and the empty vector pcDNA5/FRT-HPT-blue were transfected in the Flp-In T-REx 293 cell line (Invitrogen) together with the plasmid encoding the Flp recombinase. The Flp-In T-REx 293 cell line is derived from Human Embryonic Kidney 293 cells (HEK293; ATCC#CRL-1573), engineered to carry a site-specific integration site (FRT locus, Invitrogen). Stable pool of transfectants having undergone integration of plasmids at the single FRT locus were selected using Hygromycin B (HygroGold, Invivogen 200 µg/ml), as described (Derivery and Gautreau, 2010). Both control and BAHD1-expressing cells were grown in separate flasks, generating independent biological samples. DNA and RNA were extracted at passages P8 (HEK293-CT and HEK-BAHD1 replicate 1) and P10 (HEK-BAHD1 replicate 2). HEK-BAHD1 cells were checked by immunofluorescence and immunoblot for BAHD1 expression. Cells were grown at 37°C, in a humidified 10% CO_2 incubator, in Dulbecco's modified Eagle's medium with GlutaMAX TM (Gibco) supplied with 10% FBS (Gibco) and 200 µg/ml Hygromycin (HygroGold, Invivogen).

2 Supplementary Figures and Tables

2.1 Supplementary Figures

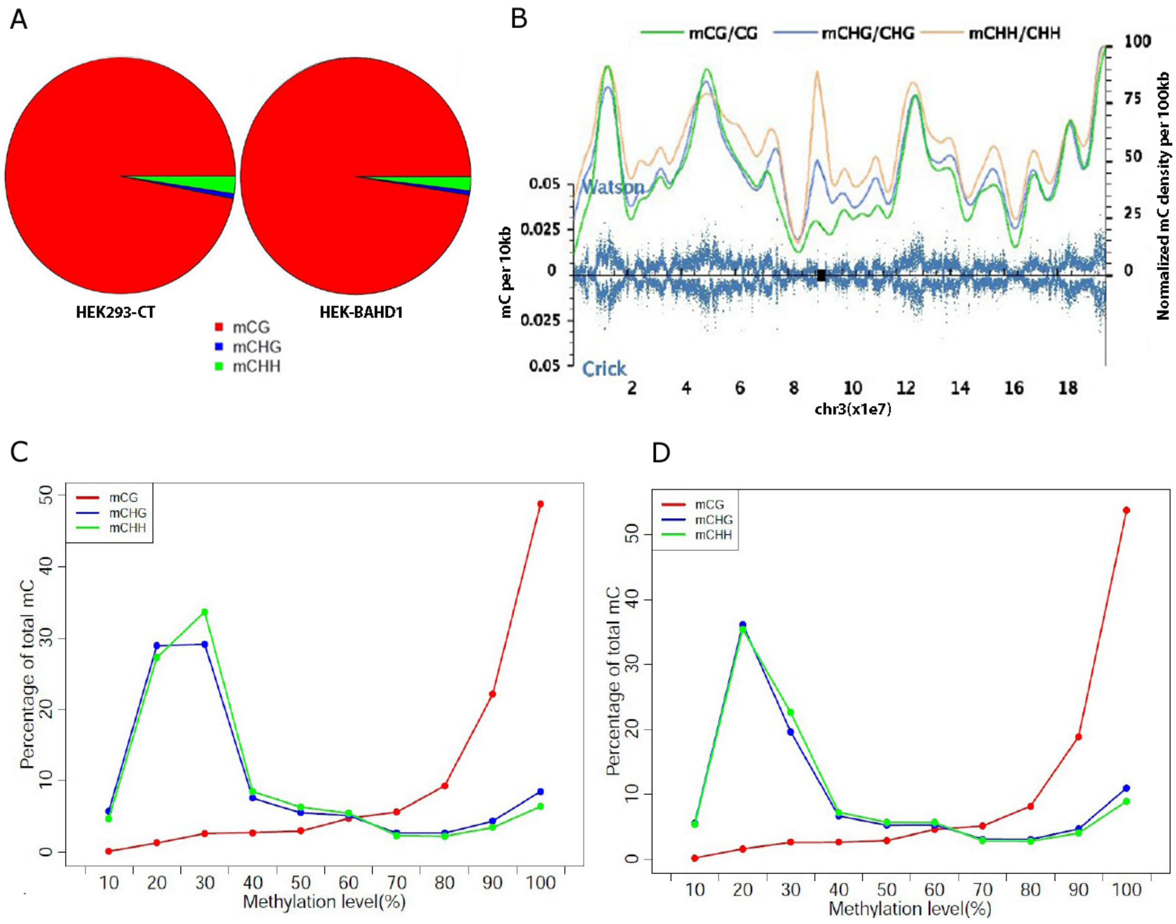

Supplementary Figure S1. Analysis of HEK-CT and HEK-BAHD1 methylomes. (**A**) % of repartition of mCG, mCHG and mCHH in HEK-CT and HEK-BAHD1 DNA. **B.** Density (per 10kb windows) of mC on the DNA strands throughout chromosome 3 for HEK-BAHD1 (black rectangle: centromere). (**C-D**) Distribution of mC per methylation level in the mCG, mCHG or mCHH context for **(C)** HEK-CT and (**D**) HEK-BAHD1. Methylation level (x-axis) is plotted against the fraction of mC for each sequence context.

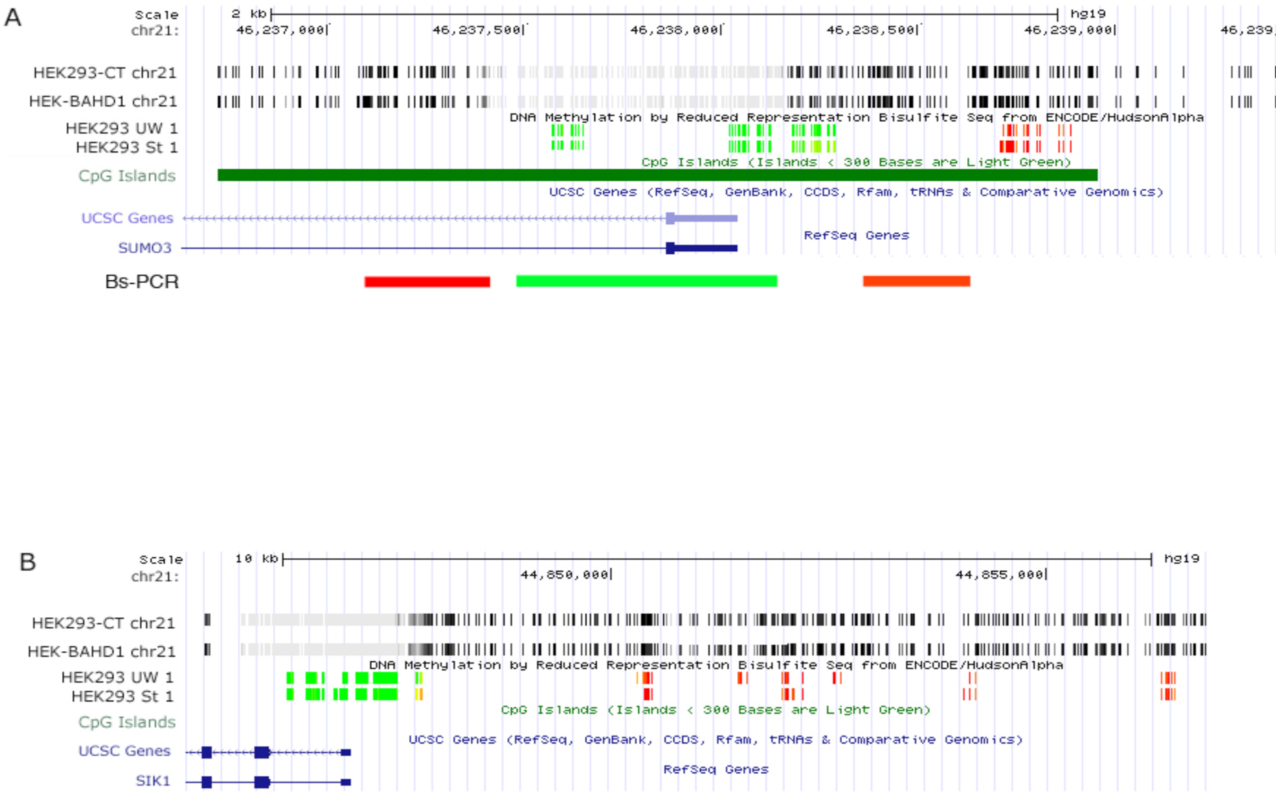

Supplementary Figure S2. Methylation profile of two control genes. Tracks are as visualized by the UCSC genome browser at the *SUMO3* **(A)** or *SIK1* **(B)** locus, as studied in Zhang et al., 2009. The tracks are as follows: BS-seq of HEK-CT and HEK-BAHD1 (this work), HAIB Methyl RRBS replicate 1 of HEK293 St and HEK293 UW1 (from ENCODE/HudsonAlpha), CpG islands, UCSC genes, RefSeq genes. Regions characterized by bisulfite PCR (BS-PCR) in HEK293 cells (Zhang et al., 2009) are squared in red (hypermethylation) and green (hypomethylation). The grey scale shows levels of methylation light (low) to dark (high).

2.2 Supplementary Tables

Supplementary Table S1. Top 80 transcription regulator binding sites overlapping with BAHD1-specific hypermethylated DMRs. This table reports results of the overlap between the BS-seq hyper-DMRs and the ENCODE TFBS cluster track (wgEncodeRegTfbsClusteredV3), representing Transcription Factor ChIP-seq Clusters V3 (161 targets, 189 antibodies) from ENCODE. The overlap is listed per TFBS, representing the top 80 regulators whose sites are found in hyper-DMRs and the number of counts of binding sites per regulator. BAHD1 known partners (from Bierne et al., 2009 and Lebreton et al., 2011) are highlighted in red. EZH2 and STAT proteins, which may play a role in BAHD1-regulated pathways, are shown in blue.

regulator	total TFBS sites	binding sites in hypermethylated DMRs	%
SETDB1	22192	787	3.5
KAP1	26393	933	3.5
GATA2	73974	1282	1.7
STAT3	67977	1175	1.7
GATA3	55528	958	1.7
FOS	131528	2224	1.7
TCF7L2	45099	744	1.6
NFIC	38848	639	1.6
JUN	58569	929	1.6
EZH2	14817	232	1.6
EP300	138816	2167	1.6
ATF2	26026	387	1.5
ZBTB7A	25061	372	1.5
POLR2A	134939	2002	1.5
ESR1	25776	378	1.5
BCL11A	20286	294	1.4
MAFK	83990	1209	1.4
FOSL2	43443	606	1.4
MAFF	47026	655	1.4
GATA1	20292	280	1.4
STAT1	19147	264	1.4
HP1-γ	20197	276	1.4
CEBPB	146823	2002	1.4
BATF	32419	433	1.3
JUND	97391	1298	1.3
FOXM1	21667	281	1.3
FOXA1	89868	1150	1.3
CTCF	162028	2045	1.3
TEAD4	58012	727	1.3
TCF12	43577	543	1.2
MYBL2	16135	200	1.2
ARID3A	24299	298	1.2
STAT5A	16120	196	1.2
RAD21	118906	1445	1.2

IRF4	17722	214	1.2
NR3C1	35169	417	1.2
SPI1	67536	795	1.2
REST	57990	680	1.2
BCL3	22616	262	1.2
TFAP2A	16808	193	1.1
RELA	36908	423	1.1
FOXA2	40831	465	1.1
NFYB	17509	199	1.1
MEF2A	21875	246	1.1
MYC	96956	1082	1.1
EBF1	48897	543	1.1
TAL1	26211	291	1.1
RUNX3	66222	734	1.1
PML	23278	258	1.1
TFAP2C	22481	246	1.1
NR2F2	16296	176	1.1
USF1	58516	626	1.1
RXRA	19225	205	1.1
HNF4A	22362	235	1.1
HNF4G	20329	213	1
TBL1XR1	22156	231	1
SP1	48986	506	1
BACH1	13283	133	1
MAX	85942	856	1
TBP	45694	443	1
ZNF263	27758	268	1
RCOR1	49688	477	1
USF2	23708	224	0.9
RFX5	24889	235	0.9
ATF1	14850	140	0.9
SMC3	56757	515	0.9
CHD2	33643	303	0.9
ATF3	23090	206	0.9
YY1	64116	570	0.9
SIN3AK20	37412	327	0.9
BHLHE40	38799	332	0.9
HDAC2	31953	272	0.9
POU2F2	26402	223	0.8
PAX5	33067	274	0.8
CHD1	16975	139	0.8
SRF	16008	131	0.8
MXI1	36258	290	0.8
TAF1	40334	317	0.8
FOXP2	27652	216	0.8
RBBP5	19199	147	0.8

Supplementary Table S2. ChrX genes down-regulated in HEK-BAHD1 when compared to HEK-CT cells. List of 72 genes repressed in response to BAHD1 overexpression. The “activity” status of these genes on the inactive X chromosome, either “always inactive” or “heterogeneous”, is based on Zhang et al., 2013. The presence of hypo-methylated DMRs in HEK-BAHD1 BS-seq replicates is indicated by “+”.

name	description	status	Hypo-DMRs in replicate 1	Hypo-DMRs in replicate 2
CD99	CD99 molecule			
KAL1	Kallmann syndrome 1 sequence		+	+
MID1	midline 1 (Opitz)		+	+
PRPS2	phosphoribosyl pyrophosphate synthetase 2	Heterogeneous	+	+
GPM6B	glycoprotein M6B		+	+
PIR	pirin (iron-binding nuclear protein)	Heterogeneous	+	+
CA5B	carbonic anhydrase VB, mitochondrial			
PHKA2	phosphorylase kinase, alpha 2 (liver)	Heterogeneous	+	+
GPR64	G protein-coupled receptor 64		+	+
MAP3K15	mitogen-activated protein kinase kinase kinase 15	Heterogeneous	+	+
PRDX4	peroxiredoxin 4	Always_inactive	+	+
SAT1	spermidine	Heterogeneous	+	+
APOO	apolipoprotein O	Heterogeneous	+	+
KLHL15	kelch-like 15 (Drosophila)	Always_inactive	+	+
PDK3	pyruvate dehydrogenase kinase, isozyme 3	Heterogeneous	+	+
GK	glycerol kinase (*GK*), transcript variant 3,	Always_inactive	+	+
XK	X-linked Kx blood group (McLeod syndrome)		+	+
SRPX	sushi-repeat containing protein, X-linked		+	+
TSPAN7	tetraspanin 7		+	+
CASK	calcium	Always_inactive	+	+
MAOB	monoamine oxidase B		+	+
TIMP1	TIMP metallopeptidase inhibitor 1	Heterogeneous	+	+
EBP	emopamil binding protein (sterol isomerase)	Heterogeneous	+	+
PCSK1N	proprotein convertase subtilisin	Always_inactive	+	+
SYP	synaptophysin		+	+
MAGED4B	melanoma antigen family D, 4B		+	
MAGED2	melanoma antigen family D, 2		+	+
APEX2	APEX nuclease (apurinic	Heterogeneous	+	+
XAGE-4	XAGE-4 protein		+	+
MSN	moesin	Always_inactive	+	+
DLG3	discs, large homolog 3 (Drosophila)	Always_inactive		+
SLC7A3	solute carrier family 7 (cationic amino acid transporter, y+ system), member 3		+	
SLC16A2	solute carrier family 16, member 2 (thyroid hormone transporter)		+	+
ZNF711	zinc finger protein 711		+	+
PCDH19	protocadherin 19		+	+
TSPAN6	tetraspanin 6		+	+

TRMT2B	tRNA methyltransferase 2 homolog B (S. cerevisiae)	Heterogeneous	+	+
CENPI	centromere protein I	Always_inactive	+	+
ARMCX2	armadillo repeat containing, X-linked 2		+	
GK3P	glycerol kinase 3 pseudogene		+	
BEX4	brain expressed, X-linked 4	Always_inactive	+	+
NGFRAP1	nerve growth factor receptor (TNFRSF16) associated protein 1	Always_inactive	+	
IRS4	insulin receptor substrate 4			
ACSL4	acyl-CoA synthetase long-chain family member 4	Always_inactive	+	+
TMEM164	transmembrane protein 164	Heterogeneous	+	+
AMMECR1	Alport syndrome, mental retardation, midface hypoplasia and elliptocytosis chromosomal region gene 1		+	+
AMOT	angiomotin	Heterogeneous	+	+
LRCH2	leucine-rich repeats and calponin homology (CH) domain containing 2		+	+
IL13RA1	interleukin 13 receptor, alpha 1	Always_inactive	+	+
ZCCHC12	zinc finger, CCHC domain containing 12		+	+
LONRF3	LON peptidase N-terminal domain and ring finger 3		+	+
ZBTB33	zinc finger and BTB domain containing 33	Heterogeneous	+	+
FAM70A	family with sequence similarity 70, member A		+	+
ODZ1	odz, odd Oz		+	+
ZDHHC9	zinc finger, DHHC-type containing 9		+	+
MBNL3	muscleblind-like splicing regulator 3	Heterogeneous	+	+
GPC4	glypican 4	Heterogeneous	+	+
GPC3	glypican 3		+	+
PLAC1	placenta-specific 1	Heterogeneous	+	+
FAM127C	family with sequence similarity 127, member C			
FAM127A	family with sequence similarity 127, member A		+	
ARHGEF6	Rac		+	+
CDR1	cerebellar degeneration-related protein 1, 34kDa			
SPANXA2-OT1	SPANXA2 overlapping transcript 1 (non-protein coding)		+	+
LDOC1	leucine zipper, down-regulated in cancer 1		+	+
IDS	iduronate 2-sulfatase	Heterogeneous	+	+
MAMLD1	mastermind-like domain containing 1		+	+
CD99L2	CD99 molecule-like 2		+	+
NSDHL	NAD(P) dependent steroid dehydrogenase-like	Heterogeneous	+	+
PNMA6C	paraneoplastic Ma antigen family member 6C			
SLC6A8	solute carrier family 6 (neurotransmitter transporter, creatine), member 8		+	+
DUSP9	dual specificity phosphatase 9	Always_inactive	+	

References

Derivery, E., and Gautreau, A. (2010). Assaying WAVE and WASH complex constitutive activities toward the Arp2/3 complex. *Methods Enzymol* 484**,** 677-695. doi: 10.1016/B978-0-12-381298-8.00033-2.

Lebreton, A., Lakisic, G., Job, V., Fritsch, L., Tham, T.N., Camejo, A., Mattei, P.J., Regnault, B., Nahori, M.A., Cabanes, D., Gautreau, A., Ait-Si-Ali, S., Dessen, A., Cossart, P., and Bierne, H. (2011). A bacterial protein targets the BAHD1 chromatin complex to stimulate type III interferon response. *Science* 331**,** 1319-1321.

Zhang, Y., Castillo-Morales, A., Jiang, M., Zhu, Y., Hu, L., Urrutia, A.O., Kong, X., and Hurst, L.D. (2013). Genes that escape X-inactivation in humans have high intraspecific variability in expression, are associated with mental impairment but are not slow evolving. *Mol Biol Evol* 30**,** 2588-2601.

Zhang, Y., Rohde, C., Tierling, S., Jurkowski, T.P., Bock, C., Santacruz, D., Ragozin, S., Reinhardt, R., Groth, M., Walter, J., and Jeltsch, A. (2009). DNA methylation analysis of chromosome 21 gene promoters at single base pair and single allele resolution. *PLoS Genet* 5**,** e1000438. doi: 10.1371/journal.pgen.1000438.
